# Role of metabolic modulator Bet-CA in altering mitochondrial hyperpolarization to suppress cancer associated angiogenesis and metastasis

**DOI:** 10.1038/srep23552

**Published:** 2016-03-22

**Authors:** Suchandrima Saha, Monisankar Ghosh, Samir Kumar Dutta

**Affiliations:** 1 Drug Development Diagnostic and Biotechnology Division, CSIR- Indian Institute of Chemical Biology (CSIR-IICB), 4, Raja S.C. Mullick Road, Kolkata-700032, West Bengal, India

## Abstract

Solid tumors characteristically reflect a metabolic switching from glucose oxidation to glycolysis that plays a fundamental role in angiogenesis and metastasis to facilitate aggressive tumor outcomes. Hyperpolarized mitochondrial membrane potential is a manifestation of malignant cells that compromise the intrinsic pathways of apoptosis and confer a suitable niche to promote the cancer associated hallmark traits. We have previously reported that co-drug Bet-CA selectively targets cancer cells by inducing metabolic catastrophe without a manifest in toxicity. Here we report that the same molecule at a relatively lower concentration deregulates the cardinal phenotypes associated with angiogenesis and metastasis. In mice syngeneic 4T1 breast cancer model, Bet-CA exhibited effective abrogation of angiogenesis and concomitantly obliterated lung metastasis consistent with altered mitochondrial bioenergetics. Furthermore, Bet-CA significantly lowered vascular endothelial growth factor (VEGF) levels and obviated matrix metalloproteases (MMP-2/9) production directly to the criterion where abrogation of autocrine VEGF/VEGFR2 signalling loop was documented. *In vitro* studies anticipatedly documented the role of Bet-CA in inhibiting actin remodeling, lamellipodia formation and cell membrane ruffling to constitutively suppress cell motility and invasion. Results comprehensively postulate that Bet-CA, a mitochondria targeting metabolic modulator may serve as an excellent candidate for combating angiogenesis and metastasis.

Cancer angiogenesis and metastasis are ostensibly paradoxical events that transform a primary tumor into systemic, metastatic life threatening disease^1^. Apoptosis resistance, angiogenesis and metastasis are often envisaged as separate, independent events but are of utmost importance when it comes to tumor progression^2^,^3^,^4^,^5^,^6^. Among the major complications associated with cancer is the phenomenology of metastatic dissemination that is essentially considered to be the final process in the progression of malignant tumors. The propensity of tumor cells to avidly consume glucose in a hypoxic environment is conceptualized as the Warburg phenotype where cells consequentially limit their energy metabolism largely to glycolysis^7^,^8^. Although no etiological implication can be drawn with oncogenesis and much remains unproven about this phenomenon, the acidic microenvironment provides hospitable conditions to facilitate angiogenesis and metastasis^9^,^10^,^11^. As tumor cells proliferate, cells localized in the lesion core are subjected to unfavorable conditions including deficient oxygen and nutrient supply^12^,^13^. This subsequently leads to the generation of hypoxia that furthers the transcription of hypoxia-inducing factor 1 (HIF-1) that regulates and activates genes implicated with angiogenesis^14^,^15^,^16^.

Hyperpolarisation of the mitochondria is a chronic feature of cancer that capacitates it to handle apoptosis and ensures insensitivity to anti-proliferative signals. This phenomenon participates in a plethora of biochemical cascades and is intricately threaded with the hallmark traits^17^. A compelling body of evidence portrays that impairment of mitochondrial membrane potential with effective mitochondria targeted drugs might obliterate features of the neoplastic phenotype^18^,^19^.

The major prerequisite for metastasis is the progression of primary tumor *in situ* to subsequent into invasive tumor. Cancer cell invasion is an extremely synchronous process yet a complex integrated circuitry that requires coordination of multiple mechanisms coherent migration^20^. The strategic rationale adopted by the cancer cells for initiating local invasion include acquisition of cell motility, increased migratory activity, constitutive actin remodeling, heightened activation of extracellular proteases and neovasculature generation to eventually orchestrate invasion promoting metastatic dissemination^21^,^22^.

Various physiological and regenerative functions triggers MMPs, a group of zinc dependent matrix degrading enzymes that alternatively play an imperative role in cell invasion and oncogenic progression. The expression of MMP-2, MMP-7 and MMP-9 is critically associated with various types of malignant tumors^23^,^24^ that allow endothelial cells to develop renewed vasculature to migrate into the tumor and concomitantly release proangiogenic factors like FGF, VEGF and TGF^25^,^26^,^27^. VEGF plays a major role in tumor angiogenesis and its expression in breast cancer is inversely correlated with patient survival^28^. Numerous human breast cancers demonstrate increased expression of vascular endothelial growth factor receptor 2 (VEGFR2) on the tumor cell as well as on the associated endothelial cells. The preeminent existence of the VEGF/VEGFR2 autocrine signalling loop facilitates resistance against chemotherapy and substantially amplifies the proangiogenic signaling pathway. Blood vessels produced within the tumors by chronically activated VEGF signal have shown to elicit capillary sprouting, excessively convoluted vessel branching and increased levels of endothelial cell proliferation and migration^29^,^30^,^31^.

We have previously established Bet-CA as an important metabolic mediator that elicits a robust anti-tumor effect both *in vitro* and *in vivo*^32^, hinting us to deduce its additional effects in disrupting angiogenesis and reversing metastatic dissemination. In this study, we have further evaluated and comprehended that Bet-CA at a relatively lower concentration, strategically inhibits angiogenesis and stalls metastasis to ultimately deliver a proficient attrition to tumor progression and concomitantly underlining the use of metabolic modulators in the rapidly expanding field of cancer therapeutics.

## Results

### Bet-CA inhibits angiogenesis by altering mitochondrial membrane potential *in vivo*

**Figure 1:**
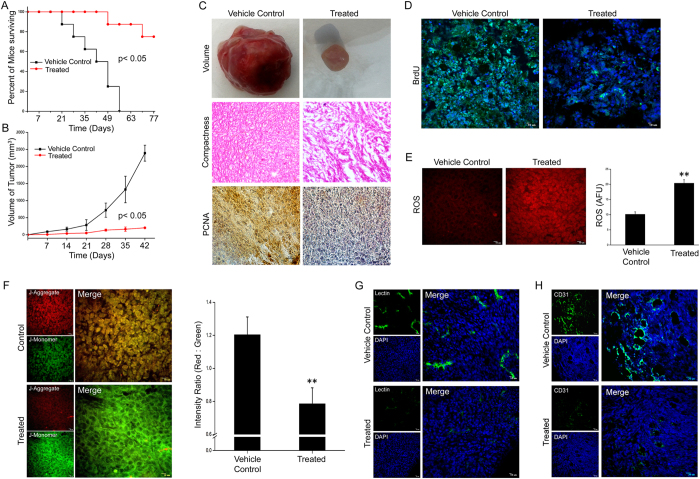
Bet-CA inhibits breast cancer angiogenesis *in vivo.* Eight tumor burdened BALB/c mice were taken for each of vehicle control and Bet-CA treated groups. (**A**) Bet-CA (1 mg/kg) administration increases mice survivability. ^∗^p < 0.05. (**B**) Graph delineates the comparative quantification and reduction of tumor growth rate in Bet-CA treated mice with respect to that of vehicle control. ^∗^p < 0.05. (**C**) Macroscopic examination demonstrates reduced tumor associated vasculature in Bet-CA treated mouse (Upper Panel). Histological analysis demonstrates less cellular density in treated sets compared to that of vehicle control (Middle Panel). Bet-CA treated animals demonstrate lower PCNA expression (Lower Panel). (**D**) Representative images present lower BrdU uptake in Bet-CA treated tumors indicating decreased cellular proliferation. (**E**) Freshly excised 4T1 tumors from Bet-CA treated sets exhibits increased levels of ROS generation compared to that of vehicle control. Graph represents quantitative estimation of ROS. ^∗∗^p < 0.01. (**F**) Micrographs illustrate that freshly excised tumors from treated mice have compromised mitochondrial membrane potential. Graph represents quantitative determination of red: green fluorescence intensity ratio. ^∗∗^p < 0.01. (**G**) Treatment depicts decreased lectin signal compared to vehicle control sets (Right Panel- enlarged view of merged images). (**H**) Representative CLSM micrographs exhibit decreased endothelial CD31 expression in Bet-CA treated 4T1 tumors. In all panels error bars represent mean ± SD.

To thoroughly elucidate the mechanistic implication of Bet-CA, we testified it’s prospective as a metabolic modulator that attenuates angiogenesis and metastasis in 4T1 breast cancer model^33^. 1 mg/kg of Bet-CA treatment significantly accentuated survival during a 77 days follow up period by causing a sustained limitation to tumor growth (Fig. 1A,B). Analogously, macroscopic examination of tumors revealed widespread tumor necrosis and vascularization in vehicle control whereas treated sets exhibited decreased tumor burden with no necrotic lesion or vasculature formation leading us to postulate the effective role of Bet-CA in restricting tumor augmentation (Fig. 1C; Upper Panel). Additionally, treatment compromises the cellular density of tumors unlike that of vehicle control where they are densely packed (Fig. 1C; Middle Panel). Thereby, data depicts that Bet-CA restricts cancer cell proliferation and to ensure this phenomenon, we strategically quantified the levels of PCNA expression and BrdU incorporation in tumors. Treatment significantly lowered PCNA expression, whereas a high level of this antigen expression was observed in vehicle control tumor tissue (Fig. 1C; Lower Panel). To further substantiate the above phenomenon *in vivo* BrdU labelling experiments were performed and immunohistochemical analysis suggests that Bet-CA treatment profoundly inhibits proliferation as sections depict low level of BrdU uptake as compared to that of vehicle control (Fig. 1D and Supplementary Fig. S1).

Previously we had reported that Bet-CA treatment integrated ROS generation and dampened mitochondrial membrane potential gradient *in vitro*^32^. To investigate whether Bet-CA expanded the same effect *in vivo*, we categorically analyzed ROS production and alterations in mitochondrial membrane potential within the tumor tissue. Treatment lead to a 2.0 fold increase in ROS generation in spite of the buffering capacity associated with the tumor microenvironment, resoundingly stating the prominent role of Bet-CA in promoting oxidative damage to the tumor (Fig. 1E). Equally importantly, to understand the role Bet-CA in mediating the potential degradation of mitochondrial polarization, tumor sections were stained with JC-1 and TMRM dye. Results reveal that Bet-CA treatment correspondingly compromised mitochondrial polarization as JC-1 stained 4T1 cells and tissue sections portrayed a significant decrease in red: green fluorescence intensity ratio when compared to vehicle control cohort (Fig. 1F and Supplementary Fig. S2). Furthermore, TMRM staining of 4T1 cells and tumor tissues showed decreased red fluorescence in treated sets factually comprehending the role of Bet-CA in degrading mitochondrial membrane potential (see Supplementary Fig. S3).

Another feature of the 4T1 tumor is its dense vascularisation and since Bet-CA effectively abrogated mitochondrial membrane potential, we postulated that treatment might simultaneously inhibit the phenotypic traits of angiogenesis. Vascular density of the tumor was quantified using endothelium specific lectin and comparative quantification clearly demonstrated decreased vessel density post treatment (Fig. 1G and Supplementary Fig. S4). Additionally, tissue sections were stained using CD31 to detect neovasculature density and results expectedly portray decreased vascularized area in treated tumor sections (Fig. 1H). Suitably, Bet-CA understandably combats and modulates the phenomenology of angiogenesis by essentially degrading the mitochondrial membrane potential and comprehensively limits the chronic displays of cancer acquired hallmark traits.

### Bet-CA inhibits pulmonary metastatic potential of 4T1 tumors

**Figure 2:**
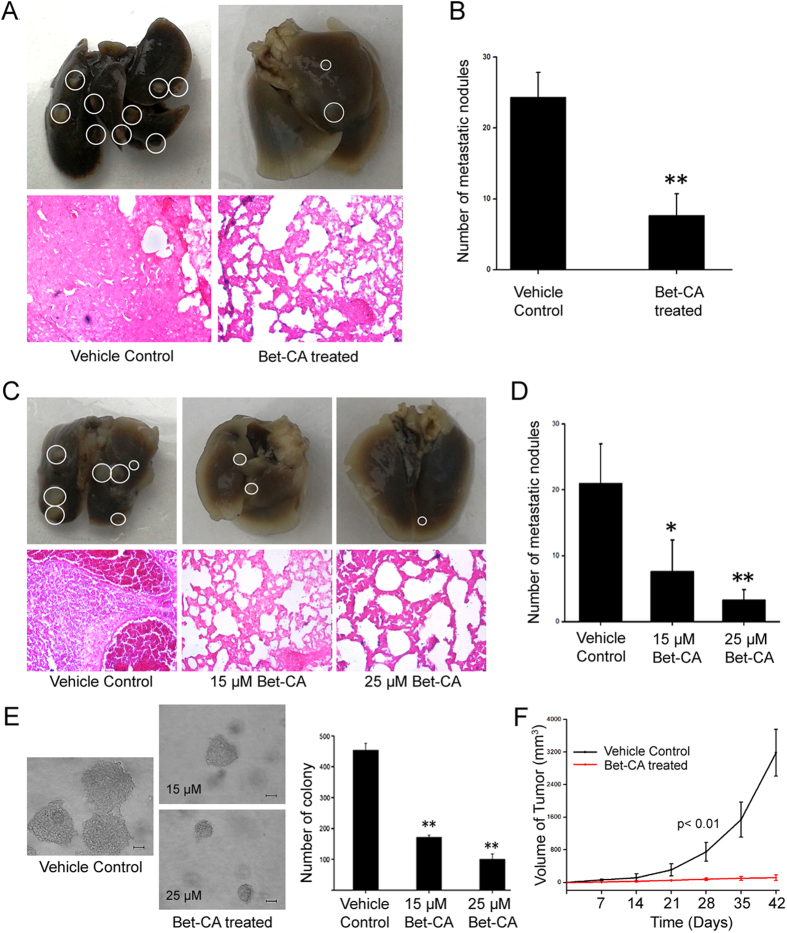
Bet-CA degrades metastatic potential *in vivo*. (**A**) Images depict that administration of 1 mg/kg of Bet-CA effectively suppresses spontaneous metastatic pulmonary nodule formation compared to vehicle control (Upper Panel). Lung sections depict the area corresponding to 4T1 lung colonization in vehicle control and treated mice (Lower Panel). (**B**) Graph represents quantification of lung metastatic nodules on both dorsal and ventral sides. ^∗∗^p < 0.01. (**C**) Representative images illustrating lung colonization potential of Bet-CA treated 4T1 cells (Upper Panel). H & E staining depict the metastatic lesion area (Lower Panel). (**D**) Corresponding comparative analysis of lesions in mice experimental metastasis model. ^∗^p < 0.05. (**E**) Bet-CA inhibits clonogenic outgrowth of 4T1 cells compared to vehicle control. Graph depicts restricted sphere formation in Bet-CA treated sets. ^∗∗^p < 0.01. (**F**) Graph represents compromised tumor initiating potential of Bet-CA treated 4T1 cells *in vivo*. ^∗∗^p < 0.01. Microscopic images were captured at 20X magnification. In all panels error bars represent mean ± SD.

Metastasis is one of the major complications of general cancer suppressive therapy due to the associated risk of recurrence and development of cancer de novo^34^. Since Bet-CA effectively outdoes with tumor growth and angiogenesis, we characterized whether it rigorously attenuates the associated traits of metastasis. 4T1 cells injected into the mammary fat pad of BALB/c mice relevantly stimulated metastasis to the lungs within four weeks, however Bet-CA effectively abrogated the spontaneity to metastasize under physiological conditions. Mice were sacrificed and lungs were analyzed to determine the number of pulmonary metastatic lesions (Fig. 2A; Upper Panel). Additionally, histological analysis of lung sections portray that treatment resulted in small, disseminated metastatic foci starkly in contrast to that of vehicle control that had extensive tumor foci area and fast growing metastases (Fig. 2A; Lower Panel). Graph showcases quantification of breast cancer nodules, where vehicle control tumors retain the self inherent ability of dissemination however, lesion formation was suppressed upon treatment (Fig. 2B) which suggestively concrete the potential of Bet-CA to limit metastatic dissemination of the tumor cells. Successful colonization into distant organ sites is a major criterion for the establishment of micro and macro metastasis. To accurately scrutinize whether Bet-CA only limits the ability of tumor cells to spread from the primary site, or if it also affects the colonization stage of metastasis, we used experimental metastasis model. Accordingly, 15 and 25 μM of Bet-CA treated 4T1 cells were administered directly into the circulation by intravenous (*i.v.*) injection to form pulmonary metastatic lesions. After 14 days, nodules were counted and lung sections were analyzed (Fig. 2C; Upper Panel). As anticipated, vehicle control cells generated large number of micro and macro metastases whereas mice with treated cells had relatively less number of metastatic lesions (Fig. 2C; Lower Panel). Comparative quantification illustrates the number of nodules in vehicle control and treated sets factually comprehending the role of Bet-CA in chronically inhibiting the crucial stages of metastasis (Fig. 2D).

Metastatic cancer cells have an inherent clonogenic potential and capability of anchorage-independent growth. To address this additional commonality associated with metastasis, soft-agar colony formation assay was employed^35^. As shown in figure, vehicle control 4T1 cells formed large tumor spheres of about 454.6 ± 10.7 μm in diameter after 14 days whereas, treatment with 15 and 25 μM Bet-CA resulted in the staggering regression of colony size to 171.2 ± 3.3 μm and 100.6 ± 8.4 μm respectively (Fig. 2E). Prompted by the observation that Bet-CA limits tumor initiation in soft agar, we examined if it also discouraged tumor initiation *in vivo* and as anticipated, 25 μM Bet-CA treated cells failed to form tumors when injected into mammary fat pad of BALB/c mice (Fig. 2F). Thus, taken together, results project the potential of Bet-CA to limit and arrest the metastatic manifestations.

### Bet-CA inhibits actin remodeling, lamellipodia formation and cell polarization

**Figure 3:**
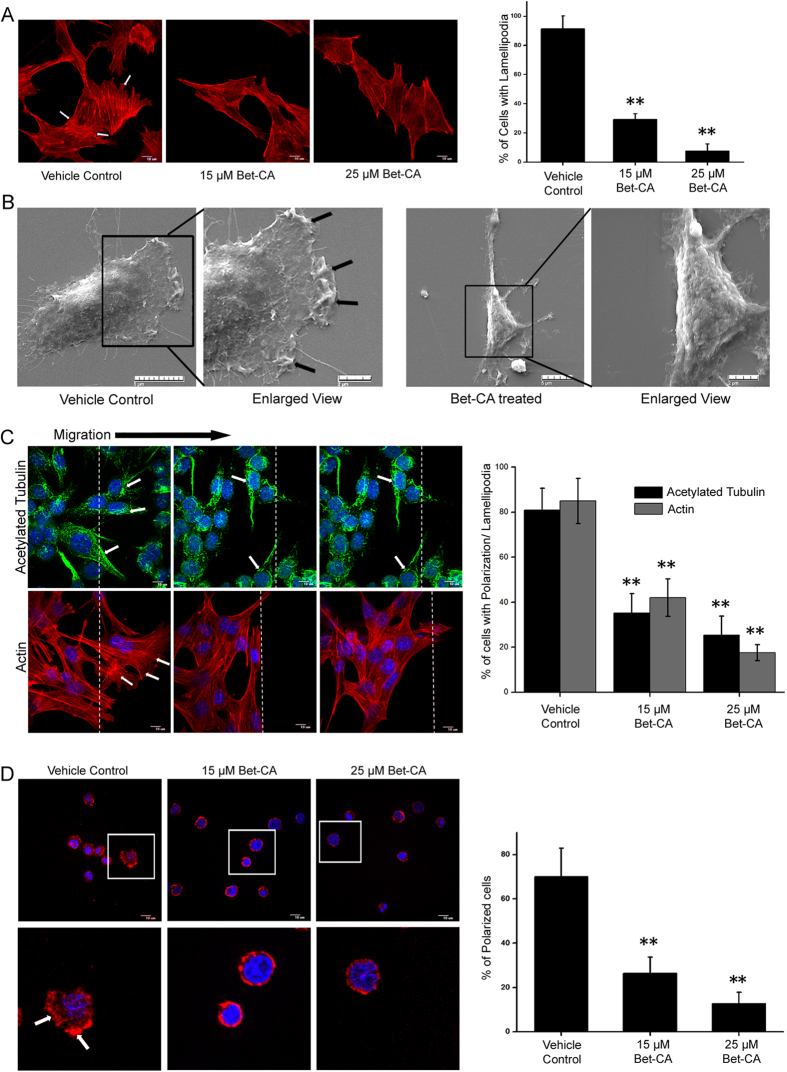
Bet-CA inhibits cytoskeletal dynamics. (**A**) Representative CLSM micrographs exhibiting inhibitory effect of Bet-CA on lamellipodia formation in 4T1 cells. Arrows indicate lamellipodia. The number of cells exhibiting lamellipodia were quantified and graphically represented. ^∗∗^p < 0.01. (**B**) Scanning EM images reveal that Bet-CA suppresses lamellipodia formation with respect to that of vehicle control. Arrows indicate membrane ruffling (Enlarged view). (**C**) Representative images portray that Bet-CA treatment restricts directional cell polarization and lamellipodia formation. Wound edge is represented by white dashed lines, MTOC and actin ruffles has been depicted by arrows. Cells were stained for f-actin (red), acetylated tubulin (green) and nucleus (blue). The numbers of polarized cells and cells with actin ruffles has been quantified, analyzed and graphically represented. ^∗∗^p < 0.01. (**D**) Bet-CA inhibits chemotactic cell polarization. Representative CLSM images depict directional actin protrusions in vehicle control that is arrested post Bet-CA treatment. Graph represents the % of polarized cells. ^∗∗^p < 0.01. (Lower Panel- enlarged view). Images were captured at 60X magnification. In all panels error bars represent mean ± SD.

Metastasis is a multistep sequential process and contains diverse temporal courses that saliently feature the phenomenologies of directional migration and therefore, we furthered our investigation to gain insights into whether Bet-CA impaired the migratory and invasive behaviors of cancer cells. Bet-CA rigorously halts F-actin assembly associated with the aggravated migratory phenotype and graph represents the quantification of F-actin expression and lamellipodia formation post 15 and 25 μM Bet-CA treatment (Fig. 3A). Actin reorganization is abruptly halted as cells adopt a radiative morphology unlike that of vehicle control (see Supplementary Fig. S5) and true to our postulation, Bet-CA disintegrated lamellipodia formation and stress fibers within the cells, ensuring important repercussion and efficient inhibition of cytoskeletal protein polymerization and remodeling. Membrane ruffling is a consistent feature associated with migration and treatment substantially arrests ruffle formation as visualized employing scanning electron microscopy (Fig. 3B). To gain further implication of Bet-CA corresponding to the factors implicated with directional cell migration, a scratch assay was performed. Treatment significantly compromised F-actin polymerization and acetylated tubulin recruitment on the migrating front to resultantly slow down microtubule turnover. Graph comparatively showcases the effect of Bet-CA in limiting cell polarization and lamellipodia formation (Fig. 3C). Cancer cells when chemotactically attracted to a particular extracellular stimulus, accentuates dynamic cell polarization and culminates in the activation of directional cell migration. In differentiated HL60 cell polarization model^36^, Bet-CA treatment thwarted cell polarization (Fig. 3D) and categorically inhibited directional cell migration and associated cytoskeletal dynamics.

### Bet-CA attenuates breast cancer cell migration and invasion *in vitro*

**Figure 4:**
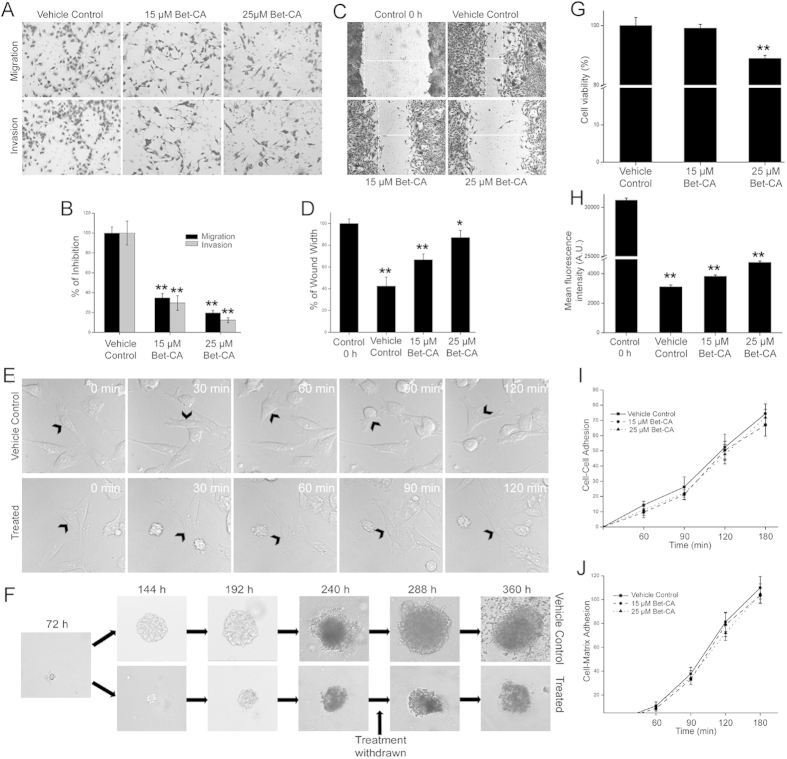
Bet-CA treatment suppresses 4T1 migration and invasion. (**A**) Bet-CA inhibits cancer cell migration (Upper Panel) and invasion (Lower Panel) in transwell assay. Cells were treated with Bet-CA for 16 h and cells on the lower sides of the chambers were stained and representative images are shown. (**B**) Graphical quantification of 4T1 cell migration and invasion with respect to that of vehicle control. **p < 0.01. (**C**) Representative images depict that treatment inhibits 4T1 cell migration in scratch healing assay. (**D**) Graphical representation of the width of the wound. *p < 0.05, **p < 0.01. (**E**) Live cell imaging of vehicle control and 25 μM Bet-CA treated 4T1 cells were done for 2 h and positions of the migrating cell at different recorded time points have been shown. Direction of cell movement is indicated by arrowheads and the field was constant throughout acquisition. (**F**) Representative images depict that Bet-CA reversibly inhibits acquisition of the invasive phenotype in 3D matrigel assay. (**G**) Viability of 4T1 cell line on 15 and 25 μM of Bet-CA treatment was analyzed using MTT assay. Bars represent % of cell viability after 24 h. **p < 0.01. (**H**) Quantitative flow cytometric analysis of the effect of Bet-CA on 4T1 cell proliferation. **p < 0.01. Cells were treated with 15 and 25 μM of Bet-CA and were assayed for adhesion (**I**) to other 4T1 cells or (**J**) to type I collagen for various time periods. Error bars represent mean ± SD.

The heterogeneous strategy of metastasis is mirrored by its persistent ability to migrate and invade and since Bet-CA inhibited cancer associated cytoskeletal dynamics, we immediately testified its potential in regulating migratory phenotypes. To this end, we employed transwell assay to determine the extent of cell migration and invasion and understandably, treatment with 15 or 25 μM of Bet-CA for 16 h significantly deregulated the ability of 4T1 cells to crawl through the transwell (Fig. 4A,B). Similarly in wound healing assay, Bet-CA significantly suppressed cell migration, displaying its effect in restricting the ability of cells to execute the initial steps of metastasis (Fig 4C,D). Additionally, using live cell imaging technique we determined that treatment essentially compromised migratory velocity in real time (Fig. 4E). Next we reflected on whether Bet-CA portrayed effective repercussion on cell invasion in 3D matrigel matrix – a mimic of *in vivo* metastatic phenomenology^37^. Treatment of 4T1 cells with 25 μM of Bet-CA disrupted the attainment of the invasive phenotype (Fig. 4F). Treatment upto 50 μM per se, did not result in cell death as after removal of Bet-CA, the tumor emboli reversed its phenotype and therefore we exclude the possibility that shrinkage of tumor sphere during treatment was an outcome of apoptosis. Additionally, to address the possibility of the plausible effect of Bet-CA on breast cancer cell viability and proliferation, MTT and CFSE dye dilution assay was performed. Results underline the fact that treatment did not hamper cell viability except restricting its proliferation (Fig. 4G,H). As derived, Bet-CA provocatively reduced tumor growth *in vivo* and obviated directional cell migration and invasion to strategically point out for the fact that anti-metastatic effects of Bet-CA are not secondary to tumor growth or proliferation. Considering the robustness of Bet-CA, we consequentially examined whether it regulated additional pro-metastatic phenomenologies of cell-cell adhesion or cell-matrix adhesion. Treatment did not modulate cell-cell adhesion nor had any significant impact upon cell-matrix adhesion (Fig. 4I,J). In aggregate, Bet-CA comprehensively inhibited the associative traits of cancer cells for mediating metastasis.

### Bet-CA suppresses paracrine angiogenic signaling *in vitro*

**Figure 5:**
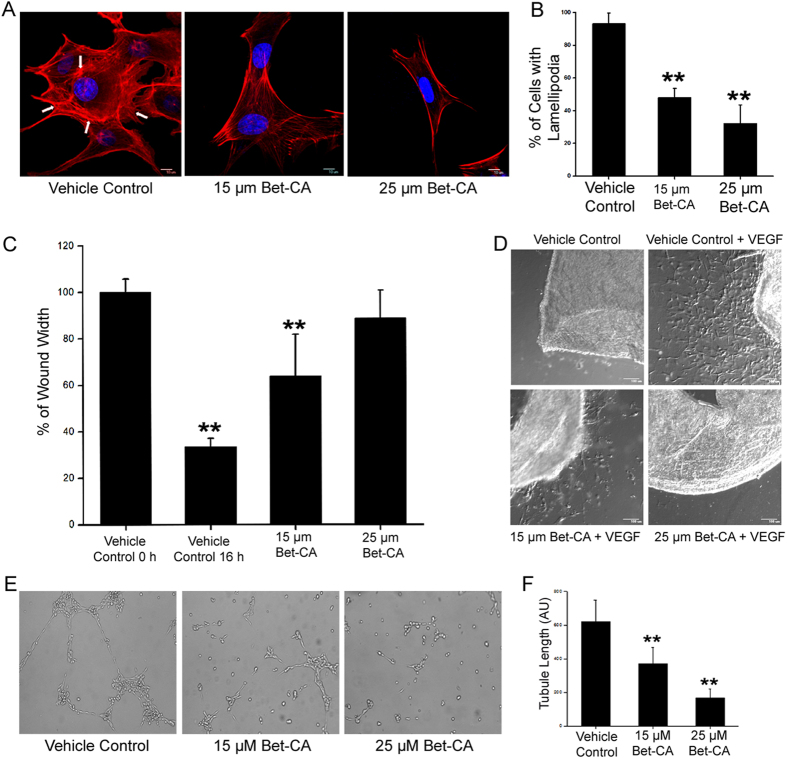
Bet-CA inhibits endothelial cell migration and associated angiogenic signaling. (**A**) Bet-CA inhibits actin remodeling and lamellipodia formation in HUVEC. Arrows indicate stress fibres and lamellipodia formation in vehicle control that is abrogated in treated sets. (**B**) Graph shows quantification of cells exhibiting lamellipodia. **p < 0.01. (**C**) Graph depicts the abrogatory potential of Bet-CA on endothelial cell migration in wound healing assay. **p < 0.01. (**D**) Bet-CA suppresses neo-angiogenesis in mice aortic ring sprouting experiment. (**E**) The effluent of Bet-CA treated 4T1 cells significantly decreases tubule formation with respect to that of vehicle control. (**F**) Graph delineates quantitation of tubule length post treatment. **p < 0.01. In all panels error bars represent mean ± SD.

Given the multitude of Bet-CA in modulating tumor vasculature formation *in vivo*, we sought to accurately triangulate important insights of its effect on paracrine angiogenic signaling. Foremost, we rationalized the aptitude of Bet-CA to effectively and efficiently deliberate a constraint on actin reorganization and lamellipodia formation of endothelial cells. As shown in Fig. 5A, there was concomitant acquisition of stress fibres and lamellipodia in vehicle control HUVECs whereas, this phenomenon typically collapsed upon Bet-CA treatment. Graph represents that treatment arrests acquired migratory traits in a dose dependent manner (Fig. 5B). Additionally, we quantified the prospect of Bet-CA in inhibiting migration by employing scratch assay technique and treatment substantially halts migration of endothelial cells similar to its role on cancer cells (see Supplementary Fig. S6). Figure 5C represents the % of migration inhibition posed by Bet-CA on endothelial cells hinting on the fact that treatment effectively fosters suppression on directional cell migration. Assertively, one of the most fundamental traits of angiogenesis is neo-vessel sprouting and to understand the complementary effect of Bet-CA on angiogenesis, we employed mouse aortic ring *ex vivo* assay^38^. Vehicle control aortic rings displayed high levels of angiogenic sprouting whereas Bet-CA significantly encompassed an inhibitory effect on cell proliferation, neo-microvessel branching and remodeling (Fig. 5D). The plasticity associated with tumor microenvironment and angiogenesis enticed us to study the direct paracrine effects of Bet-CA on tumor vasculature formation and to study this phenomenon we used endothelial tubule formation assay^39^. Resultantly, 15 and 25 μM of Bet-CA treated 4T1 supernatant significantly abrogated tube formation reflecting the effectiveness of Bet-CA in modulating and limiting tumor associated angiogenesis (Fig. 5E) and graph delineates tubule length post treatment (Fig. 5F). Hitherto, Bet-CA treatment challenges the associated angiogenic signaling circuitry within cancer and considerably obviates the phenomenon of angiogenesis.

### Influence of Bet-CA on VEGF and MMP production

**Figure 6:**
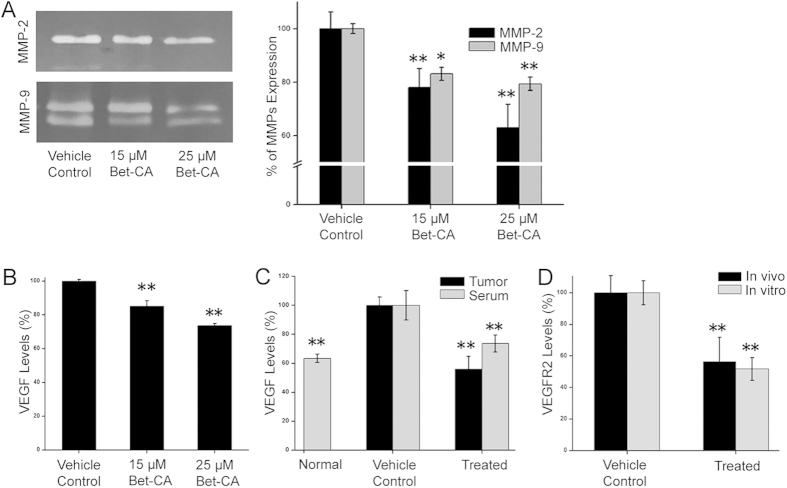
Bet-CA inhibits MMP-2/9 production and VEGF/VEGFR2 signalling axis. (**A**) Vehicle control and Bet-CA treated conditioned media of 4T1 cells were analysed using gelatin zymography. The bands were quantified and graph represents arbitrary densitometric units of gelatinolytic activity. ^∗^p < 0.05, ^∗∗^p < 0.01. (**B**) Graph represents quantification of VEGF production by vehicle control and Bet-CA treated 4T1 cells after 24 h. ^∗∗^p < 0.01. (**C**) Quantification of VEGF levels from 4T1 tumors and serum samples. Tumors and serum were isolated to determine *in situ* and circulating VEGF levels. ^∗∗^p < 0.01. (**D**) Quantitative analysis of VEGFR2 expression *in vivo* and *in vitro* in vehicle control and Bet-CA treated sets. ^∗∗^p < 0.01. In all panels error bars represent mean ± SD.

Since Bet-CA inhibits the deeply associated phenotypes of pathological angiogenesis, we suspected that it might effectively inhibit MMPs. The effect of Bet-CA on the production of MMP-2/9 in 4T1 cells was determined using gelatin zymography technique (Fig. 6A). Quantitative analysis portrays that Bet-CA coherently downregulated MMP-2 and MMP-9 production in a dose dependent manner.

MMP expression is perceived as a central regulator of metastasis and angiogenesis and since, Bet-CA potentially degrades MMP-2/9 production we supposedly determined the effect it imposes on VEGF production *in vitro* and *in vivo*. Bet-CA significantly suppressed VEGF secretion by 4T1 cells after 24 h (Fig. 6B, Supplementary Figs S7 and S8) and encouraged by the *in vitro* results, we addressed this issue *in vivo* and testified whether the therapeutic physiological concentration of Bet-CA could similarly reverse VEGF production in breast tumor bearing mice. Serum samples isolated either from non-tumor bearing mice, vehicle control tumor challenged mice or from Bet-CA treated tumor bearing mice, demonstrate that VEGF production is sufficiently suppressed from that of vehicle control and were at consistent levels with that of non-tumor bearing mice. Context dependent, we cannot rule out the possibility that decreased tumor mass of treated mice could have supposedly positively contributed to the lowering of serum VEGF levels. Therefore, tumors from vehicle control and Bet-CA treated mice were resected and analyzed for VEGF production. Quantification delineates the ability of Bet-CA to invariably limit VEGF production (Fig. 6C). Since autocrine expression of VEGFR2 is closely associated with VEGF expression, we elucidated whether it alters VEGFR2 expression levels. Quantitative analysis showcases that VEGFR2 expression level is downregulated in 4T1 tumors as well as in cells upon treatment (Fig. 6D, Supplementary Figs S8 and S9) advocating for the fact that Bet-CA rigorously impacted upon the VEGF/VEGFR2 autocrine signaling axis. In aggregate, Bet-CA modifies MMP production and fosters oncogenic suppression by thwarting the VEGF/VEGFR2 signaling axis.

### Bet-CA halts migration of compact bone derived mesenchymal stem cells (CBDMSCs)

**Figure 7:**
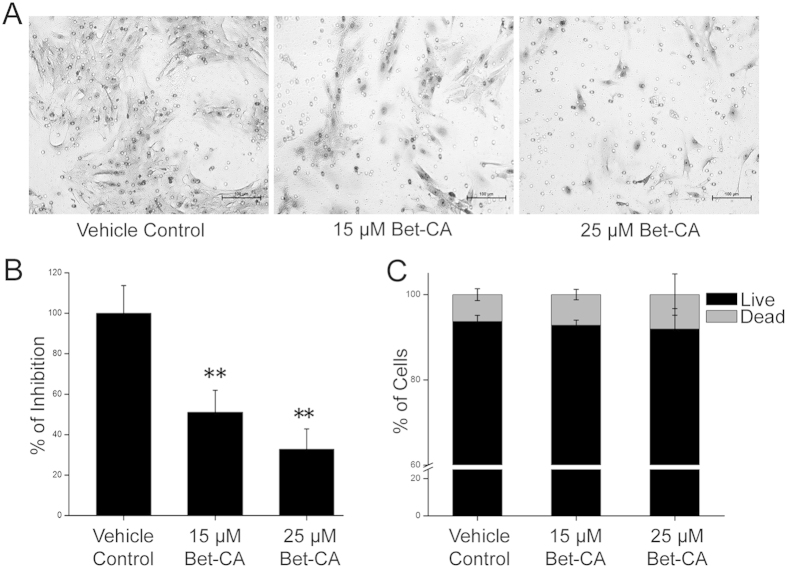
Bet-CA inhibits CBDMSC migration. (**A**) Bet-CA significantly suppresses CBDMSCs’ migration in a dose-dependent manner. (**B**) Graph depicts % of cell recruitment. ^∗∗^p < 0.01. (**C**) Quantification of apoptosis induced by Bet-CA treatment for 24 h using flow cytometry. In all panels error bars represent mean ± SD.

VEGF is one of the most prominent factors involved in the recruitment of bone marrow derived cells (BMDCs) that contributes to angiogenesis by enriching the microenvironment of primary tumor and therefore might facilitate metastatic tropism. BMDCs further differentiate into tumor associated cells (for example tumor associated fibroblasts) and replenish the tumor with angiogenic factors^40^,^41^. A major component of the stroma is cancer associated fibroblasts (CAFs) that are tumor promoting and assists tumor progression. Since, Bet-CA lowered VEGF secretion, affected the VEGF/VEGFR2 signalling loop both *in vivo* and *in vitro* and decreased VEGF levels in the media of 4T1 cells in a dose dependent manner, we further scrutinized whether it affected the recruitment of mesenchymal stem cells (mMSC) from the bone marrow to the tumors. CBDMSCs were isolated and characterized based on immunophenotyping (see Supplementary Fig. S10)^42^. Since Bet-CA considerably decreased VEGF levels in 4T1 cells and tumors, we subsequently analyzed whether it affected the migration potential of mMSCs *in vitro*. Cells were plated in the top chamber and vehicle control and Bet-CA treated 4T1 cell conditioned complete medium was used in place of the attractant in the bottom chamber. Recruitment of mMSCs was effectively restrained and quantitation elucidates the effectiveness of Bet-CA in halting mouse mesenchymal stem cell (mMSC) migration (Fig. 7A,B). Furthermore, to decipher whether the effect imposed by Bet-CA on mMSCs was strictly limited to cell migration, flow cytometry was employed to assess cell death. As anticipated, treatment did not induce apoptosis in the cells (Fig. 7C), convincingly suggesting the attribution of Bet-CA in arresting the factors contributing to angiogenesis and metastasis.

## Discussion

Successful manipulation of cancer requires strategies that must be evaluated for their effect not only on tumor growth but also in restricting metastasis and angiogenesis, as these processes help in modeling the microenvironment and leads to attainment of aggressive cancer outcomes^2^. Therefore, it might be ideal to consider therapeutic agents that target pivotal signaling hubs and consequentially provide a more durable approach to mitigate cancer. Warburg phenotype is intimately associated with the metabolic signatures of cancer cells that are inturn regulated by the mitochondria implying that these phenomena can contribute directly to the hallmark traits of cancer. Bet-CA can selectively and synergistically subvert mitochondrial apoptosis in neoplastic population and has been crucial in obliterating toxic manifestations, encouragingly addressing the problems associated with chemotherapy.

Therefore, the inhibition of the alleged events of metastasis and angiogenesis by Bet-CA that affects the mitochondria might be an attractive and more interesting than targeted inhibitors^43^,^44^. The justification being that signaling pathways are restricted more proximally by altering the resilient mitochondria that simultaneously presents less opportunity for tumor to escape given the vast majority of perturbations and alterations a cancer cell is capable of^45^. Thus, reversing the metabolic signaling signature of the mitochondria might be a substantial and logical approach to selectively inhibit angiogenesis and metastasis. Given the momentum that mitochondria targeting metabolic modulators are fast paced attaining a position in the field of cancer metabolics, our work factually demonstrate that Bet-CA dynamically attenuates tumor growth by not only reversing metastasis but also by inhibiting the associated phenomenology of angiogenesis.

To drive the expansion of Bet-CA’s potential in the smeared territories of angiogenesis and metastasis, 4T1 syngeneic mice breast cancer model was used. Convincingly, treatment with 1 mg/kg of Bet-CA increased mice survivability that is possibly the prime aim of any therapy. Results indicate Bet-CA critically abrogated tumor growth and further analysis demonstrates that tumor associated vasculature development was essentially diminished upon treatment. Moreover, results depict that tumors exposed to treatment exhibited heightened levels of intracellular ROS and degraded hyperpolarized mitochondrial manifestations provocatively indicating that Bet-CA provides less opportunity for the tumor to escape by recruitment of other metabolic pathways. In analogy with the potential of Bet-CA in altering the mitochondrial polarization, we sought to carry out angiogenesis specific studies in breast tumors. A significant reduction of tumor vessels with Bet-CA treatment was noted as positive reinforcement of lectin perfusion displayed lower levels of signal in treated animals. In regard, we further performed CD31 staining of tumors and as anticipated, treatment significantly decreased vasculature enriched areas within 4T1 tumors prospecting the role of Bet-CA in deliberating a benefit as it increases survivability and at the same time restricts angiogenesis by posing a threat to cancerous mitochondria.

The phenomenon of metastasis is dependent on angiogenesis and since Bet-CA provides a closure on tumor associated vasculature generation, it would possibly attenuate metastatic dissemination. Bet-CA treatment significantly thwarted metastatic lesion development and appreciably exhausted the colonization potential of the cells as envisaged from spontaneous and experimental metastatic model. Furthermore, Bet-CA obviated self renewal and clonogenic growing potential of 4T1 cells comprehending the potential application of Bet-CA in reciprocating the complex process of metastasis. To thoroughly understand the phenotypic alterations of tumor cells associated with metastasis and angiogenesis upon Bet-CA treatment, we delineated its mechanistic effect on the strategies adopted by cancer cells to eventuate metastatic dissemination. Treatment inhibited reorganization and polymerization of F-actin and also suppressed microtubule organization centre (MTOC) formation to eventually compromise with the core regulatory network of directional cell migration. Furthermore, Bet-CA arrested cell migration, invasion and limited acquisition of metastatic phenotype in 3D matrigel assay. Additionally, it plays the role of anti-angiogenic mediator as it compromised cytoskeletal regulatory dynamics, suppressed neovasculature formation in mice aortic ring and equally importantly, restricted tubule formation in spite of the presence of cancerous effluent, suggestively displaying the potential of Bet-CA in reversing angiogenesis.

It is widely envisaged that MMP production is essential for cell to degrade the basement membrane and consequence metastatic dissemination. Bet-CA significantly lowered MMP-2/9 production *in vitro* and from a mechanistic point of view, our study prospectively depicts that anti-angiogenic effect of Bet-CA is related to VEGF antagonism at two separate levels. At one level, VEGF production is reduced *in vitro*, as experiment evidently display diminished level of VEGF secretion on treatment and this phenomenon was evidenced *in vivo* where serum and tumor VEGF levels were lowered in Bet-CA treated mice. At another level, it inhibits the expression of VEGFR2 on tumors compromising the autocrine VEGF/VEGFR2 signaling axis. Thus, Bet-CA abrogated the activity of pro-angiogenic mediator VEGF, supposedly pointing out for the fact that it is a potential therapeutic candidate that reverses the aspects associated with carcinogenic hallmarks.

Aggressive tumor outcomes are a consequence of tumor invasiveness and formation of distal metastasis that ultimately leads to patient death. Overwhelmingly, majority of the chemotherapeutic agents that are used alone or in combination with other systemic agents for the treatment of human malignancies results in toxicity in due course. We deliberated earlier that co-drug Bet-CA, a metabolic modulator, exhibits antineoplastic activity both *in vitro* and *in vivo* against several cancers and is particularly attractive because of its milder toxicity profile and therefore has a theoretical advantage as a chemosensitizer. In this perspective, we present a logical and attractive approach to establish that Bet-CA halts metastasis and angiogenesis by mounting a demonstrable degradation of mitochondrial polarization that further inhibits MMP-2/9 production and associated VEGF/VEGFR2 signaling loop. The ability of metabolic modulator Bet-CA to act as a potential anticancer agent that can concomitantly interfere with cancer development at several points by suppressing mitochondrial function, may open a new therapeutic window in the rapidly expanding chapter of metabolic modulators in cancer therapy. Conclusively, future development of drugs specifically destabilizing the mitochondrial bioenergetics of the cancer cell, essentially non-toxic, could manifest a high clinical value. Such molecules could be combined with the current chemotherapeutic regimen or surgical approaches to have a profound therapeutic impact in the clinical setting.

## Methods

### Chemicals and Reagents

RPMI 1640, Opti-MEM, α-MEM, M199 medium, PenStrep, 3-[4,5-dimethylthiazol-2-yl]-2,5- diphenyltetrazoliumbromide (MTT), 4′,6-diamidino-2-phenylindole (DAPI), 5,5′,6,6′-tetrachloro-1,1′,3,3′- tetraethyl benzimidazolylcarbocyanine iodide (JC-1), Cell-Rox Deep Red reagent, rhodamine-phalloidin, CellTrace CFSE cell proliferation kit, fetal bovine serum (FBS), type I collagen, collagenase II and dispase were obtained from Life Technologies (Carlsbad, CA, USA). Anti-VEGFR2, FITC labeled anti BrdU, PE labeled anti- CD29, CD31, CD44, CD86 and CD105 antibodies were purchased from eBioscience (San Diego, CA, USA). Acetylated tubulin antibody was purchased from Abcam (Cambridge). Mouse VEGF antibody was purchase from Santa Cruz (USA). 8.0 μm pore transwell PET membranes and Matrigel were obtained from BD Biosciences (Bedford, MA). Mouse VEGF ELISA kit, was obtained from ThermoFisher Scientific (Waltham, MA, USA), Mouse VEGF was purchased from PeproTech (Rocky Hill, USA), FITC-conjugated Ricinus Communis Agglutinin I was purchased from Vector Laboratories (Burlinghame, CA, USA). Tetramethylrhodamine methyl ester perchlorate (TMRM), and all other reagents were purchased from Sigma (St. Louis, Missouri, USA).

### Cell Culture

4T1, MDA-MB-231 (breast cancer), HL-60 (acute promyelocytic leukemia) cells and mMSC (mouse mesenchymal stem cells) were cultured in RPMI 1640 or α-MEM medium supplemented with 10% FBS (fetal bovine serum) and 1% pen/strep. HUVEC (human umbilical vein endothelial cells) were a kind gift from Kaustubh Dutta (University of Nebraska Medical Center, Nebraska, USA) and cultured in Lonza EGM-2 media. Cells were cultured at 37 °C, 5% CO_2_ and were used within 10 passages for the experiments.

### 4T1 syngenic mice model

All experiments were performed in accordance with the national guidelines and as recommended by the animal ethics committee of CSIR-Indian Institute of Chemical Biology (Registration No. # 147/ 1999/ CPCSEA). All possible efforts were made to minimize the animals’ suffering and to reduce the number of animals used. The institutional animal care committee of CSIR-Indian Institute of Chemical Biology reviewed and approved the animal experimentation protocols. Female BALB/c mice (6 to 8 weeks), used in this study were received from the animal facility centre of CSIR-IICB and maintained under laboratory conditions (12: 12, dark: light cycle) with standard pellet diet and water ad libitum.

1 × 10^5^ 4T1 cells were injected in the upper fourth inguinal fat pad of female virgin BALB/c mice to develop spontaneous metastases model. Seven days after cell implantation, mice were randomized equally into two group containing 8 mice each. Vehicle or Bet-CA was injected intratumorally at a dose of 1 mg/kg according to the dosing schedule. Tumor size was measured weekly with calipers to assess tumor volume (mm^3^). At indicated times, animals were sacrificed, tumors and lungs were excised.

For determining pulmonary colonization and tumor initiation potential, 1 × 10^5^ 4T1 cells were grown in matrigel with or without Bet-CA for 7 days. The matrigel was washed with PBS and digested by incubation with dispase at 37 °C for 2 h. Cell aggregates were trypsinized for 5 minutes at 37 °C to obtain single cell suspension. 1 × 10^5^ cells each of vehicle control and Bet-CA treated cells were administered via lateral tail vein or were inoculated in the upper fourth inguinal fat pad of female virgin BALB/c mice. To determine the colonization potential, animals were sacrificed and lungs were analyzed after 14 days. For determining the tumor initiation potential, tumor volume (mm^3^) was assessed weekly.

### Fluorescence immunocytochemistry

Cells were seeded on coverslips, incubated at 37 °C, 5% CO_2_ and treated as mentioned. The coverslips were washed twice in PBS, fixed with 4% (w/v) paraformaldehyde for 20 min at room temperature, permeabilized with 0.2% Triton X-100 for 15 min on ice and blocked using 1% BSA in 0.2% Triton X-100 for one hour at room temperature. Cells were incubated with antibodies against acetylated tubulin, VEGF and VEGFR2 (1:200) overnight at 4 °C and with secondary antibody (1:1000) (Alexa Fluor-488 conjugate or Alexa Fluor-555) in 1% BSA at room temperature for 2 h. For detection of actin fibers, cells were incubated with rhodamine labeled phalloidin for 20  min. Nucleus was stained using DAPI. Images were captured under confocal laser scanning microscope (CLSM), processed using Andor iQ2 software and analyzed using ImageJ.

### Fluorescence immunohistochemistry

Mice were sacrificed, lungs and tumors were dissected and 8 μm thick non-consecutive sections were cut using SHANDON Cryotome E (Thermo Electron Corporation). To visualize tumor compactness and detect pulmonary metastatic lesions standard haematoxylin and eosin (H&E) staining was performed. Immuno-staining for proliferating cell nuclear antigen (PCNA) was performed using mouse biotinylated monoclonal anti-PCNA primary antibody, goat anti-mouse biotin-conjugated secondary antibody and the signals were visualized with peroxidase-diamino-benzidine and counterstained with haematoxylin. For immunofluorescent staining with antibodies to VEGF, VEGFR2 and CD31, sections were permeabilized with 0.2% Triton X-100 for 15 min on ice, blocked using 1% BSA in 0.2% Triton X-100 for one hour at room temperature and were incubated with the respective antibodies (1:200) overnight at 4 °C. Sections were then incubated with anti-mouse secondary antibody (1:1000) (Alexa Fluor-488 conjugate or Alexa Fluor-555) at room temperature for 2 h. Nucleus was stained using DAPI. Images were obtained randomly from 10 fields per section, (2–3 sections per mouse) in CLSM processed using Andor iQ2 software and analyzed using ImageJ.

### Lectin perfusion assay

Mice were heparinized using 1000 IU of heparin per kg and after 20 min, 5 mg/kg of FITC-conjugated *Ricinus communis* Agglutinin I was injected in mice via the lateral tail vein. After 30 min of lectin circulation, mice were euthanized, tumors excised and fixed in 10% neutral buffered formalin. Cryo-blocks were prepared according to standard protocol and 8 μm thin cryo-sections were obtained using Shandon Cryotome E (Thermo Electron Corporation). Nucleus was stained with DAPI and imaged using CLSM and processed using Andor iQ2 software. Lectin signal was quantified using ImageJ software.

### Mitochondrial membrane potential and ROS estimation from freshly excised 4T1 tumors

20 μm thick sections were dissected from vehicle control and treated tumor tissues using microtome and were imaged from ten random fields after incubation with JC-1, TMRM or CellROX Deep Red Reagent for 30 min at 37 °C. Images from 10 fields were obtained randomly from each mouse and all the groups were handled in an identical manner, allowing the same time for incubation with the dyes. The image acquisition was done using CLSM, processed in Andor iQ2 software and mean fluorescence intensities were calculated using ImageJ software.

### Cell polarisation assay

HL-60 cells were differentiated with 1.3% DMSO for 7 days, and resuspended in HBSS medium containing 1.8% human serum albumin. Cells were treated at indicated concentrations and incubated for 16 h, followed by stimulation with 100 nM fMLP for 3 min. Then cells were fixed with 4% paraformaldehyde for 20 min at room temperature, permeabilized with 0.2% Triton X-100 for 20 min, and blocked with 1% BSA in 0.2% Triton X-100 for 1 h and labeled with rhodamine labeled phalloidin for 20 min and counterstained with DAPI to detect cell nucleus. All images were obtained using CLSM at 60X magnification.

### Tube formation assay

The reorganization step of angiogenesis was modeled employing tube formation assay. 96-well plates were pre-coated with 70 μl of the Matrigel basement membrane matrix per well for 4 hr at 37 °C. HUVEC were suspended in serum-free M199 medium and plated on Matrigel at a density of 2 × 10^4^ cells per well. Bet-CA was added at indicated concentrations and after 8-hr incubation at 37 °C, phase-contrast images of the endothelial tubes were obtained using Nikon Eclipse T*i* microscope at 20X magnification.

### Aortic ring assay

The thoracic aortas were dissected from 6-week old BALB/c mice and the fibro-adipose tissue around the aortas was carefully removed. The aortas were cut into 1 mm long ring and serum-starved overnight. The next day the rings were embedded into 50 μl collagen type I gel in 96-well plate and carefully fed with 150 μl of Opti-MEM culture medium supplemented with 2.5% (vol/vol) FBS, penicillin-streptomycin and 30 ng/ml of VEGF with or without different concentrations of Bet-CA. The growth medium was initially changed first on 3^rd^ day and subsequently every alternate day. DIC images were obtained on day 8 under CLSM at 20X magnification.

### 3D-matrigel assay

Wells of a 24-well dish were precoated with 200 μl of undiluted phenol-red free Matrigel (10.2 mg/mL; BD Biosciences, San Jose, CA). Cells were harvested, washed three times with PBS, and diluted to a concentration of 1 × 10^4^ per well in a volume of 200 μl. Cells were mixed with 100 μl of undiluted ice-cold Matrigel for a ratio 2:1, and laid over the bottom layer. After gelling, complete culture media was added and changed every 2 to 3 days. Morphology was assessed at various time points by Nikon Eclipse T*i* microscope at 20X magnification.

### Compact Bone Marrow Derived Mesenchymal Stem Cell (CBDMSC) isolation

Mouse CBDMSCs were isolated according to the protocol (33). In brief, 2 week BALB/c mice were killed, epiphyses removed from femurs and tibias, marrow flushed out and excised into chips of approximately 1–3 mm^3^. These chips were suspended in complete α-MEM with 1 mg/ml of collagenase II and digested for 2 h in a shaking incubator at 37 °C. The bone chips were plated and after 5 days CBDMSCs were obtained. Cells were characterized by immnophenotyping and in terms of differentiation potential into adipocytes and osteoblasts. Cells within 3–8 passages were used for *in vitro* experimentations.

### Statistical analysis

All experiments were performed in triplicates and data are representative of three independent experiments. For significance testing, unpaired two sample t-tests was used for comparisons between individual treatments, ANOVA with post hoc analysis for comparison among three or more groups and Kaplan Meier was used for survival studies. *p < 0.05 and **p < 0.01 were considered statistically significant. All statistical analyses were performed using OriginPro 8 SR0 software.

## Additional Information

**How to cite this article**: Saha, S. *et al*. Role of metabolic modulator Bet-CA in altering mitochondrial hyperpolarization to suppress cancer associated angiogenesis and metastasis. *Sci. Rep.* **6**, 23552; doi: 10.1038/srep23552 (2016).

## Supplementary Material

Supplementary Information
