# Supplementary material for: Role of metabolic modulator Bet-CA in altering mitochondrial hyperpolarization to suppress cancer associated angiogenesis and metastasis
Source: Sci Rep. 2016 Mar 22;6:23552. doi: 10.1038/srep23552 (PMC4802328; doi:10.1038/srep23552)
Supplement: Supplementary Information [file srep23552-s1.pdf]

Electronic Supporting Information

Role of metabolic modulator Bet-CA in altering mitochondrial hyperpolarization to suppress cancer associated angiogenesis and metastasis

Suchandrima Saha[§], Monisankar Ghosh[§], Samir Kumar Dutta*

Drug Development Diagnostic and Biotechnology Division, CSIR- Indian Institute of Chemical Biology (CSIR-IICB), 4, Raja S.C. Mullick Road, Kolkata-700032, West Bengal, India.

([§]S.S. and M.G. contributed equally to this work).

[*]Author for correspondence: Tel No.: - +91-33-24995701, Fax: - +91-33-24735197, +91-33-24723967, Email: - samirkdutta@iicb.res.in, somir_india@yahoo.com

Methods

BrdU Incorporation Assay. 10^6 4T1 cells were injected into the 4th inguinal mammary fat pad of BALB/c mice. Vehicle control and 1 mg/kg of Bet-CA treated mice were sacrificed after 21 days and BrdU was continually injected in each mouse at 500 µg/day for 3 days before sacrifice. Tumors were dissected, excised and 8 µm thick non-consecutive sections were obtained using SHANDON Cryotome E (Thermo Electron Corporation). Sections were incubated in 1M HCl for 10 min on ice followed by 2M HCl for 10 min at room temperature, then 20 min at 37°C. Immediately after acid incubation steps the samples were incubated in citrate-phosphate buffer (pH-7.4) for 10 min at room temperature. Sections were washed in PBS, permeabilized with 0.2% Triton X-100 for 15 min on ice, blocked using 1% BSA in 0.2% Triton X-100 for one hour at room temperature and were incubated with FITC conjugated anti-BrdU antibodies overnight at 4ºC. Sections were counterstained with DAPI and images were obtained randomly at 20X from 10 fields per section, (2–3 sections per mouse) in CLSM and processed using Andor iQ2 software.

Detection of mitochondrial membrane potential using JC1 and TMRM. Mitochondrial depolarization was assessed by CLSM (Andor) and flow cytometry using JC-1 and TMRM dyes. Cells were cultured on coverslips overnight, treated with 15 and 25 µM of Bet-CA for 16 h at 37˚C, washed twice with PBS and incubated with 10 µg/ml of JC-1 at 37˚C for 15 min. Finally cells were washed and analysed by CLSM at 40X. For FACS, 15 and 25 µM of Bet-CA pre-treated cells were harvested, suspended in PBS and incubated with 25 ng/ml of TMRM at 37°C for 15 min. Stained cells were washed twice, suspended in PBS and analysed using BD LSRFortressa II flow cytometer.

CFSE Cell Proliferation Assay. Cells were harvested in PBS and 10 µM of CFSE was added and incubated for 15 minutes at 37°C. Cells were centrifuged and further the cell pellet was resuspended fresh pre-warmed complete medium. Further the cells were incubated for another 30 minutes rewashed and *in vitro* cell cultures were set. Cells were harvested and analyzed using a flow BD LSRFortressa II flow cytometer with 488-nm excitation and data using analyzed FACS DIVA 6.2 software.

Cell Viability Assay. MTT assay was employed to estimate cell viability. 10^4 cells of 4T1, MDA-MB-231 were seeded onto flat bottom 96-well plates and incubated at 37˚C in 5% CO_2. Cells were treated with 15, 25 µm or without Bet-CA and incubated for 72 h. 20 µl of 5 mg/ml MTT labelling reagent was added and absorbance of the samples were measured at 570 nm using Thermo MULTISKAN FC plate reader.

Transwell migration and invasion assay. Transwell migration and invasion assay was performed according to standard protocol. Briefly, 5 $x10^4$ cells were suspended in serum-free media with or without treatment and plated onto 8.0 micrometer pore transwell PET membranes. 500 µl of medium containing 5% serum and growth supplements were added to the bottom well. After 16 hr incubation at 37°C, cells were fixed and stained with giemsa. Non-migrating cells on the upper surface of the filter were removed, and stained cells that migrated to the lower side were photographed from ten random fields at 20X magnification using Nikon Eclipse T*i* microscope (20X).

Wound Healing Assay. 2×10^4 of cancer or endothelial cells were seeded into collagen-coated 24-well plates. After reaching confluence, cell monolayers were scratched with a sterile 200 µl pipette tip to obtain a “wound”. The media and dislodged cells were aspirated, and replaced by fresh media with or without treatment. After 16 hr incubation at 37°C with 5% CO_2, cells were fixed, stained with giemsa and photographed using Nikon Eclipse T*i* microscope (20X) from ten

random fields. The width of wounded cell monolayers in images was measured and the rates of migratory rates were calculated using ImageJ software.

Cell- matrix adhesion assay. 4T1 cells were grown on 60-mm plates and were treated with vehicle control or Bet-CA for 16 h and simultaneously other 60-mm plates were coated overnight at 37°C with 10 mg/ml type I collagen for 24 h. Next, the cells on each plate were suspended and plated on the collagen-coated 60-mm plates. After various incubation periods, the nonadherent cells were removed by agitation of the plate followed by washing once with DMEM. The number of adhered 4T1 cells was stained using giemsa and thirty random fields were counted for each plate.

Cell-cell adhesion assay. The assay was performed as described for the cell-matrix adhesion assays, except the other 60-mm plates were covered with a 100% confluent monolayer of 4T1 cells instead of collagen.

Western Blot Analysis. Cell pellets were resuspended in NP40 cell lysis buffer (1% NP40, 50 mM Tris (pH 8.0), 150 mM NaCl, 2 mM EGTA, 2 mM EDTA, protease inhibitors containing 10 μg/ml aprotinin, 5 μg/ml pepstatin, 10 μg/ml leupeptin, and 50 μg/ml PMSF, 50 mM NaF, 0.1 mM $NaVO_4$), left on ice for 30 minutes and centrifuged. The protein concentrations of the supernatant were determined using Bradford protein estimation assay kit. A total of 50 μg protein was separated using SDS-PAGE and subjected to immunoblotting using primary antibodies against VEGF, VEGFR2 and β-actin and the later was used as loading control. Bands were detected using Azure c400 (azure biosystems).

Gelatin Zymography. Aliquots of vehicle control and treated medium conditioned by 4T1 cells were analyzed by gelatin zymography in 0.1% gelatin-10% acrylamide gels. 20 μl aliquots of appropriately diluted media were loaded without reduction on gels. After electrophoresis, gels were washed with 2.5% Triton X-100 to remove SDS and incubated in incubation buffer (50 mM Tris HCl, 0.15 M NaCl, 10 mM $CaCl_2$) for 24 h. Further the gels were incubated in Coomassie Brilliant Blue solution for 2 h and destained for 1 h. Variations in MMP-2/9 concentrations was calculated from using ImageJ software.

Supplementary Figures

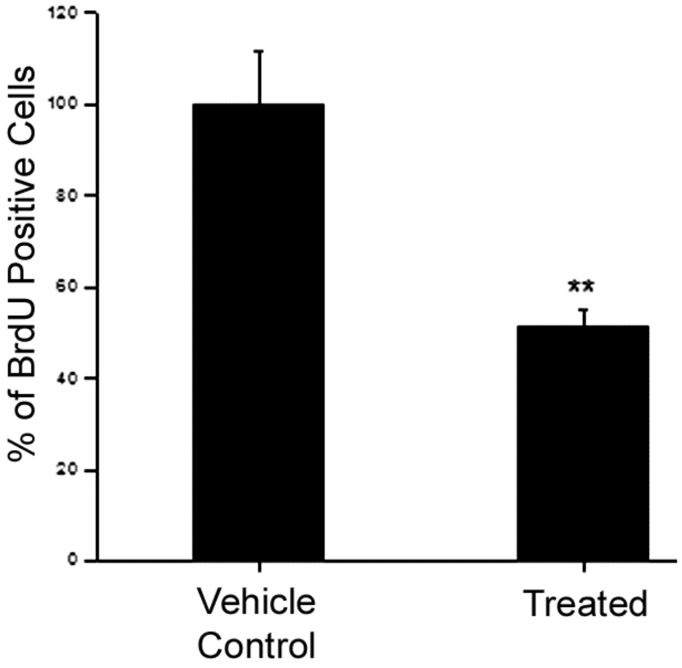

Supplementary Figure S1 | Graph represents quantification of % of BrdU positive cells *in vivo*. **p < 0.01.
In all panels error bars represent mean ± SD.

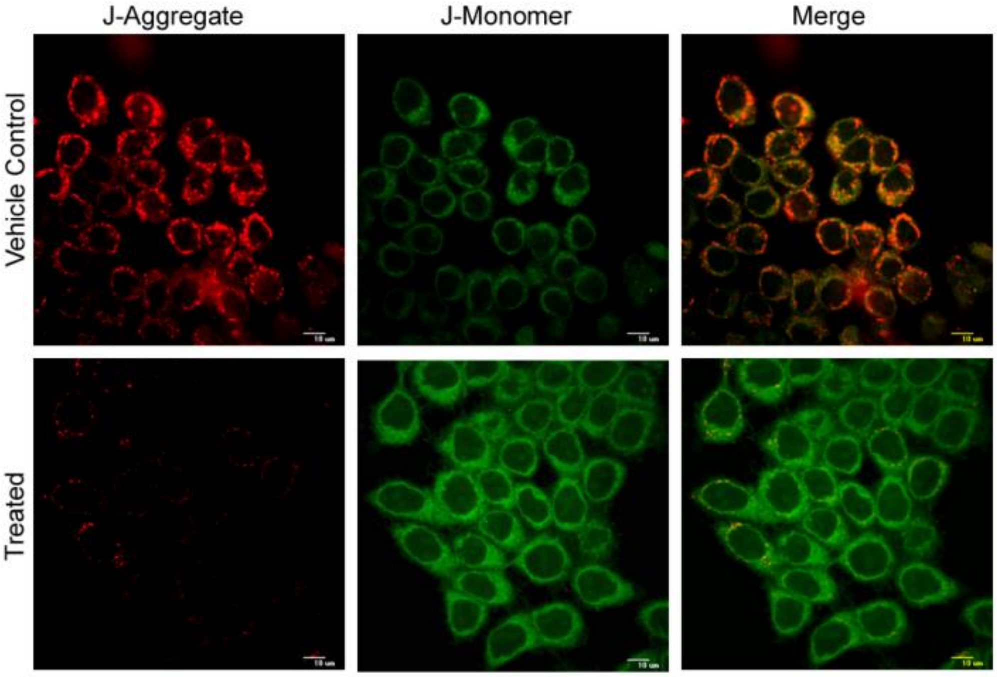

Supplementary Figure S2 | Bet-CA alters mitochondrial membrane potential *in vitro*. Representative images depict JC-1 staining pattern in vehicle control and 25 µM Bet-CA treated sets after 16 h.

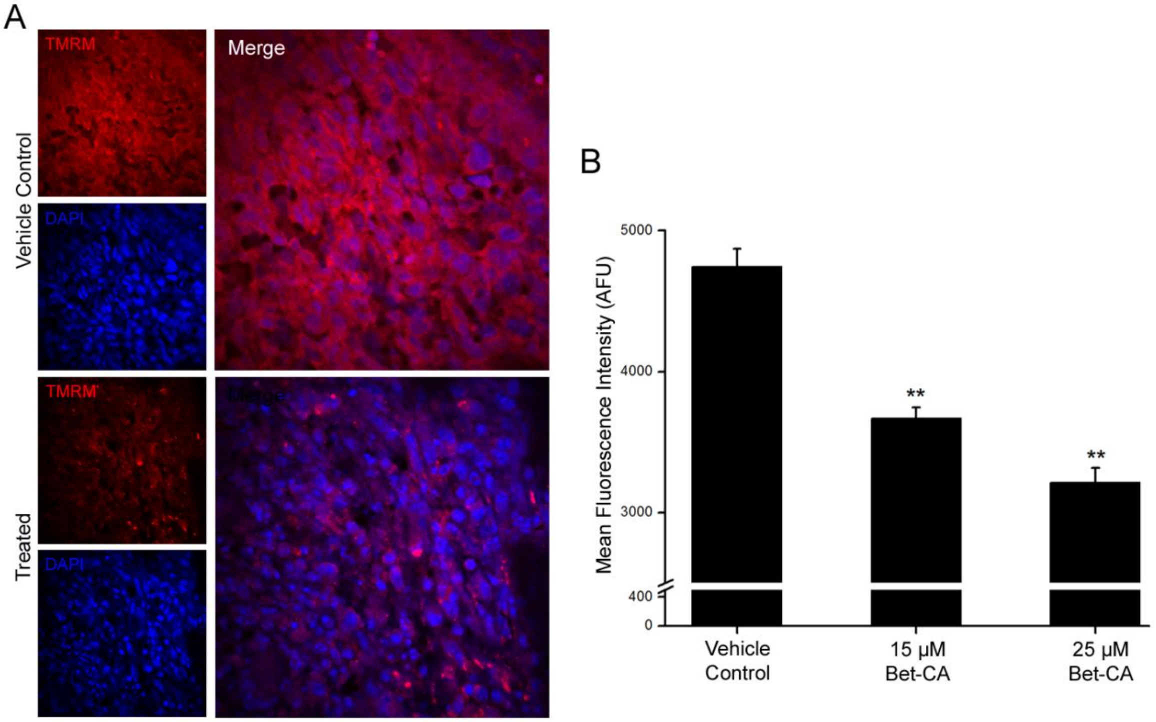

Supplementary Figure S3 | Bet-CA degrades cancerous mitochondrial hyperpolarization as analyzed using TMRM (A) Freshly resected tumors were sectioned, stained and mitochondrial potential was analyzed. Representative micrographs indicate evident decrease in red fluorescence intensity in 1 mg/kg Bet-CA treated tumors (right panel- enlarged view). (B) Flow cytometric analysis depicting quantitative decrease in red fluorescence intensity in 4T1 cells. **$p < 0.01$.

Error bars represent mean ± SD.

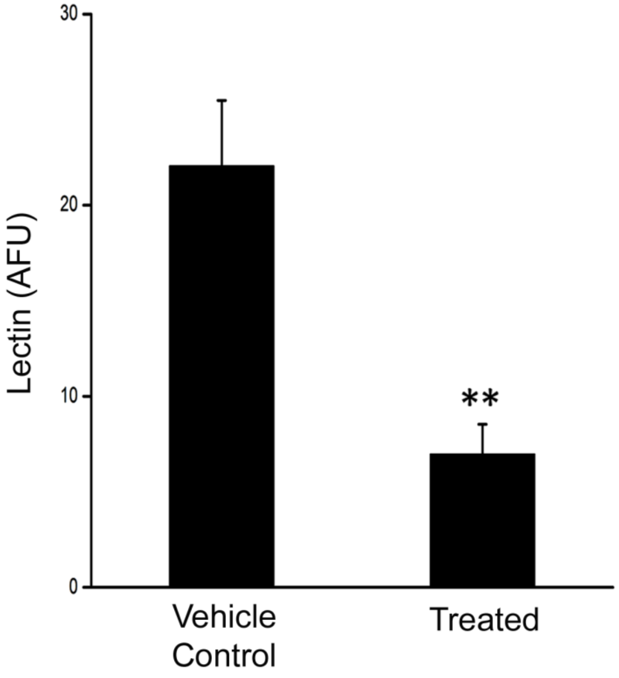

Supplementary Figure S4 | Graph represents quantification of lectin signal intensity. **$p < 0.01$.
In all panels error bars represent mean ± SD.

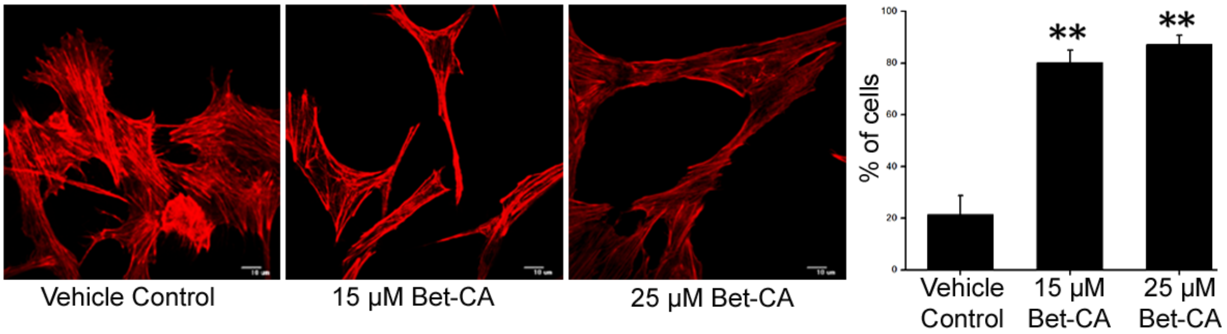

Supplementary Figure S5 | Bet-CA constrains directional cell polarization. Representative CLSM images demonstrating radialized morphology of 4T1 cells post Bet-CA treatment. Graph depicts quantitation of cells exhibiting multiangularradiative morphology in %. **$p < 0.01$.
Error bars represent mean ± SD.

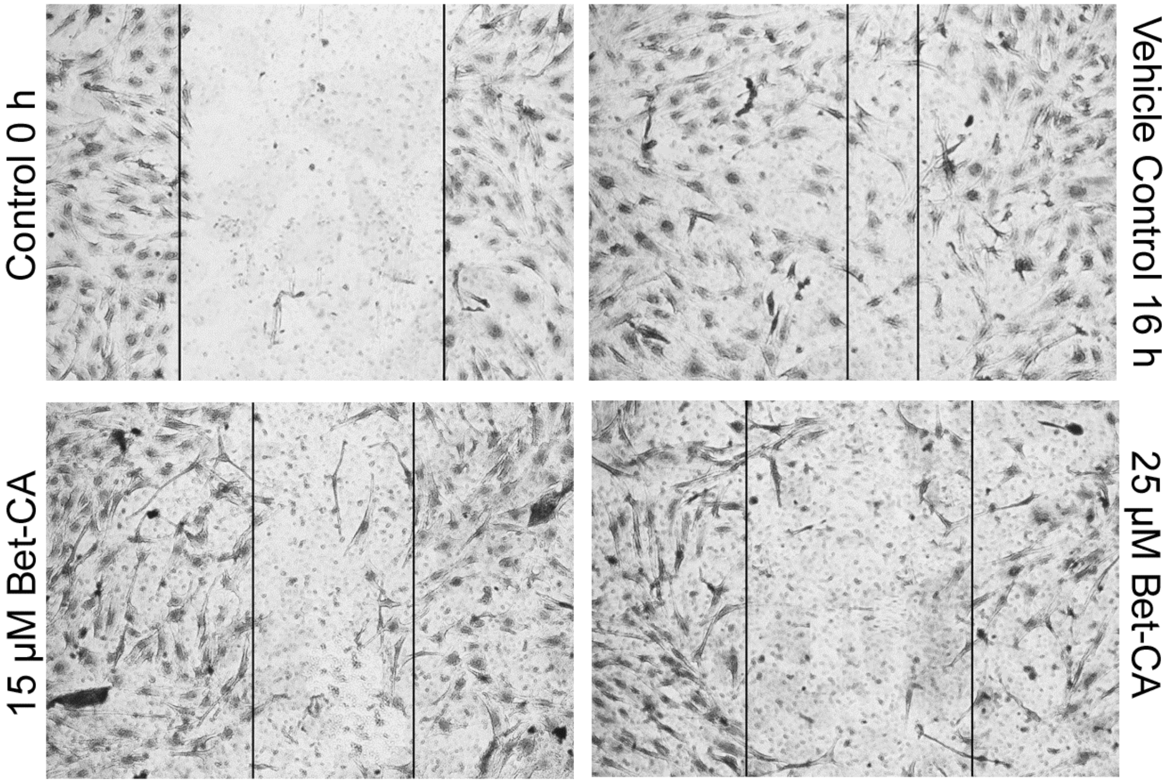

Supplementary Figure S6 | Bet-CA abrogates endothelial cell migration in a wound healing assay. Representative images of the wound width measured from five random fields are shown.

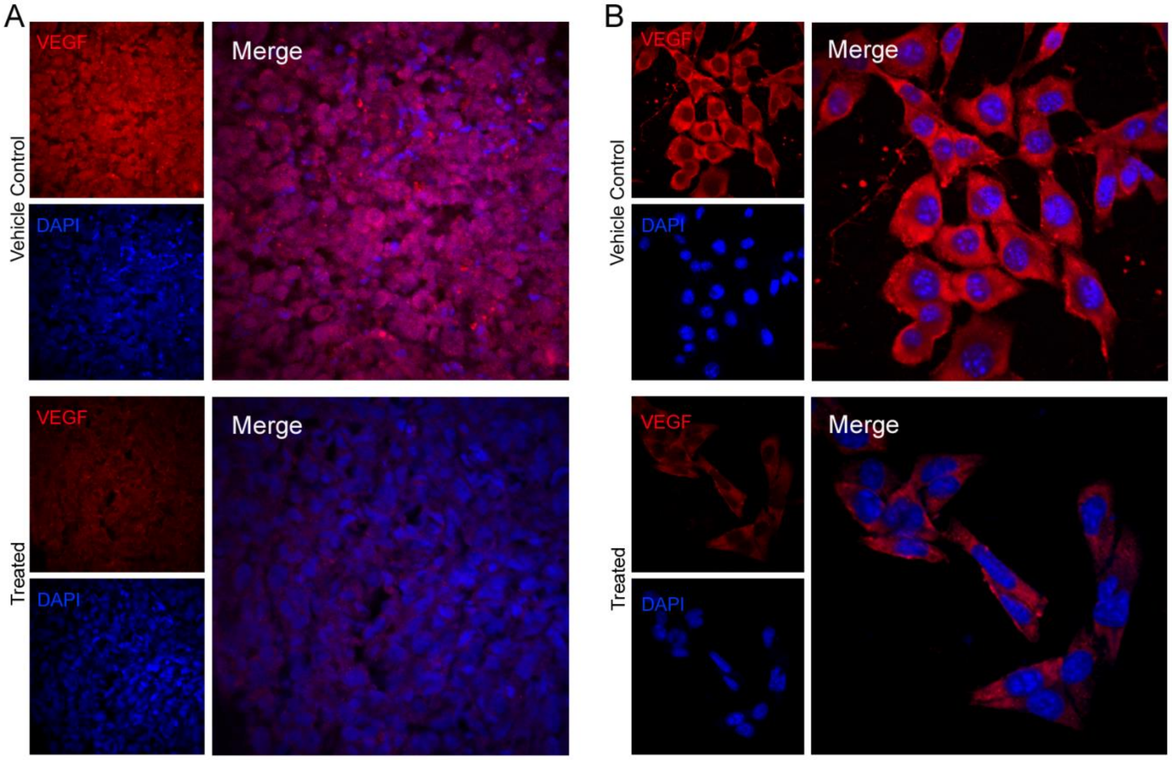

Supplementary Figure S7 | Bet-CA attenuates VEGF production *in vivo* and *in vitro.* CLSM images showcasing VEGF levels in 4T1 (A) tumors and (B) cells.

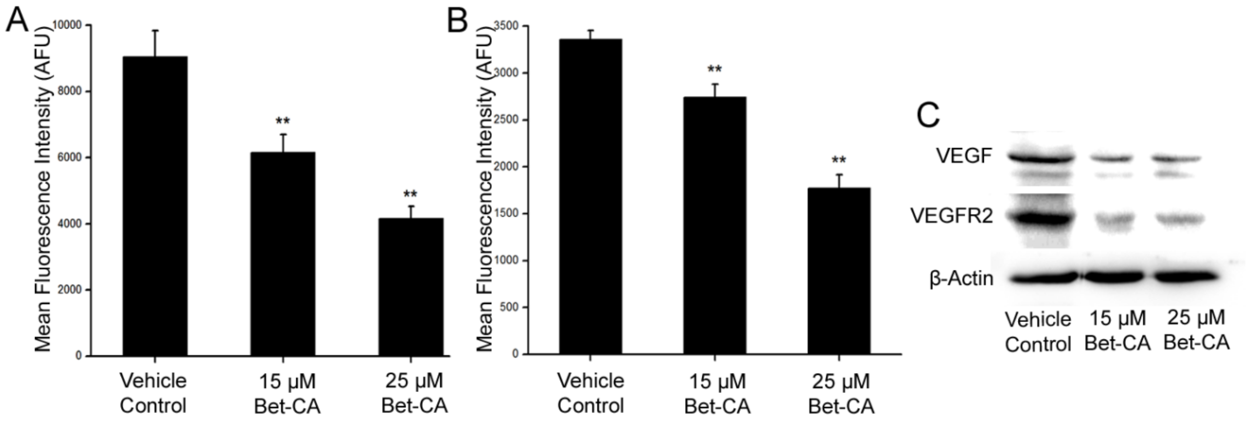

Supplementary Figure S8| Bet-CA affects VEGF/VEGFR2 signalling loop. Flow cytometric analysis depict decreased in the level of (A) VEGF and (B) VEGFR2 expression. $^{**}p < 0.01$. (C) Bet-CA treated 4T1 cells were subjected to immunoblotting for determination of VEGF and VEGFR2 expression where β-actin was used as loading control.

Error bars represent mean ± SD.

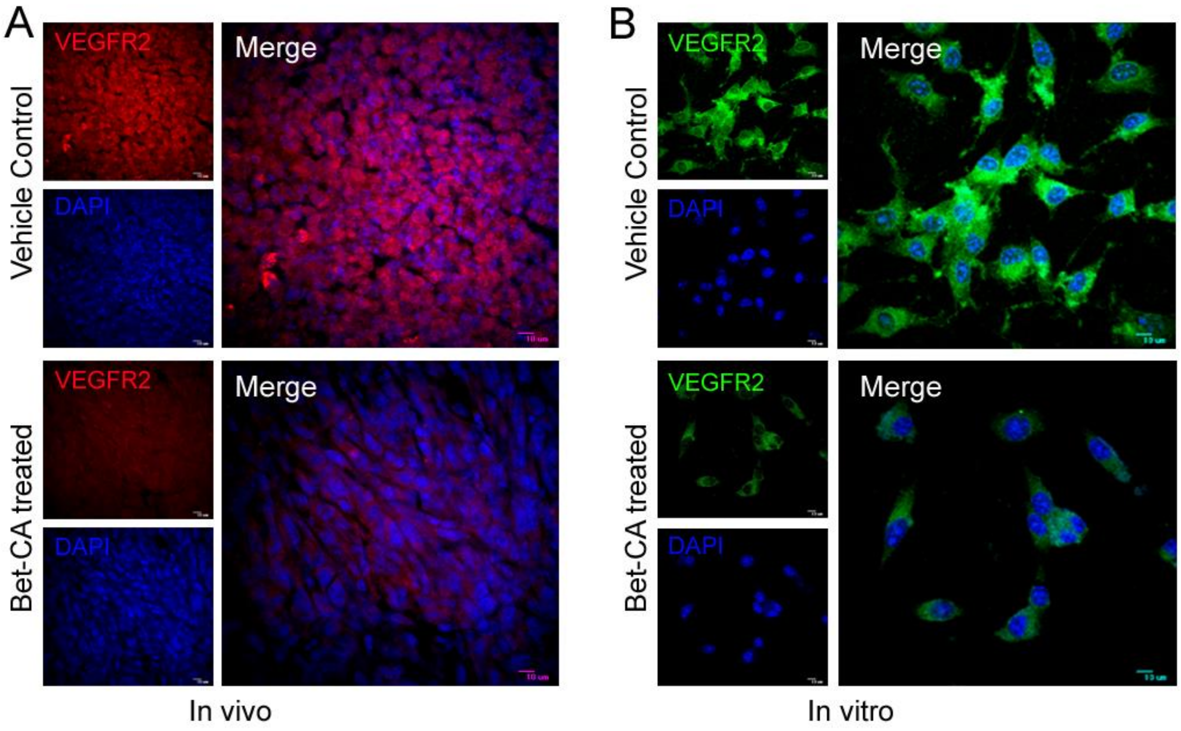

Supplementary Figure S9 | Bet-CA obviates VEGFR2 expression *in vivo* and *in vitro*. Representative CLSM images demonstrating VEGFR2 levels in 4T1 (A), tumors and (B) cells.

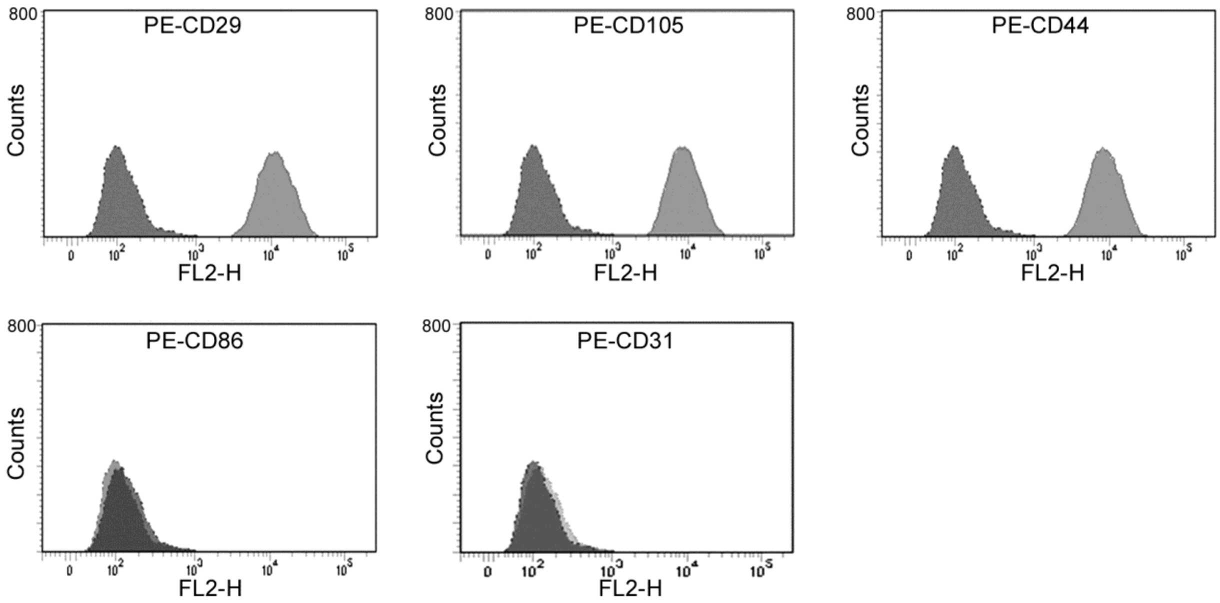

Supplementary Figure S10 | Characterization of CBDMSCs'. mMSCs' were harvested by trypsin digestion and stained with fluorescein isothiocyanate phycoerythrin (PE)-conjugated anti-mouse CD29, CD31, CD44, CD86 and CD105 antibody for immunophenotypic characterization using flow cytometric analysis. Expression profile depicts that cells were homogenously positive for mesenchymal markers CD29, CD105 andCD44; and negative for co-stimulating molecule CD86; and endothelial cell marker CD31.
